# Gene Expression Profiling of *Neospora caninum* in Bovine Macrophages Reveals Differences Between Isolates Associated With Key Parasite Functions

**DOI:** 10.3389/fcimb.2019.00354

**Published:** 2019-10-15

**Authors:** Marta García-Sánchez, Laura Jiménez-Pelayo, Pilar Horcajo, Javier Regidor-Cerrillo, Esther Collantes-Fernández, Luis Miguel Ortega-Mora

**Affiliations:** ^1^Saluvet, Animal Health Department, Faculty of Veterinary Sciences, Complutense University of Madrid, Madrid, Spain; ^2^Saluvet-Innova, Faculty of Veterinary Sciences, Complutense University of Madrid, Madrid, Spain

**Keywords:** *Neospora caninum*, bovine macrophages, isolates, virulence, transcriptome, host-parasite interactions

## Abstract

Intraspecific differences in biological traits between *Neospora caninum* isolates have been widely described and associated with variations in virulence. However, the molecular basis underlying these differences has been poorly studied. We demonstrated previously that Nc-Spain7 and Nc-Spain1H, high- and low-virulence isolates, respectively, show different invasion, proliferation and survival capabilities in bovine macrophages (boMØs), a key cell in the immune response against *Neospora*, and modulate the cell immune response in different ways. Here, we demonstrate that these differences are related to specific tachyzoite gene expression profiles. Specifically, the low-virulence Nc-Spain1H isolate showed enhanced expression of genes encoding for surface antigens and genes related to the bradyzoite stage. Among the primary up-regulated genes in Nc-Spain7, genes involved in parasite growth and redox homeostasis are particularly noteworthy because of their correlation with the enhanced proliferation and survival rates of Nc-Spain7 in boMØs relative to Nc-Spain1H. Genes potentially implicated in induction of proinflammatory immune responses were found to be up-regulated in the low-virulence isolate, whereas the high-virulence isolate showed enhanced expression of genes that may be involved in immune evasion. These results represent a further step in understanding the parasite effector molecules that may be associated to virulence and thus to disease traits as abortion and transmission.

## Introduction

*Neospora caninum* is an apicomplexan parasite that is one of the primary infectious causes of bovine abortion worldwide (Dubey et al., 2007). To survive, proliferate and transmit, this parasite has evolved mechanisms that modulate host cells according to parasite requirements, ensuring long-term survival not only of the parasite but also of the host cell (Hemphill et al., 2006). Because the immune response generated by the infection is highly associated with the control and pathogenesis of neosporosis (Almería et al., 2017), modulation of immune cell functions may be essential for parasite survival, tissue dissemination, and transplacental transmission in the host.

Recent *in vitro* studies carried out by our group have shown the ability of *N. caninum* to grow into bovine monocyte-derived macrophages (boMØs) and shown isolate-dependent differences regarding parasite cell cycle and the cellular response to infection. Specifically, the highly virulent isolate Nc-Spain7 showed higher invasion, proliferation and capacity to survive in boMØs than the low-virulence isolate Nc-Spain1H. In addition, Nc-Spain1H-infected boMØs exhibited a higher proinflammatory response (García-Sánchez et al., 2019). The boMØ transcriptome was found to be drastically regulated by *N. caninum*, which increased the expression of genes involved in pathogen recognition (e.g., TLR2, TLR3, and TLR9), NF-κB signaling pathway and proinflammatory cytokine and chemokine genes that elicit a Th1 response. *Neospora caninum* infection also restrained lysosome activity and apoptosis and modified cell metabolism. The results of the study also suggested that the high-virulence isolate Nc-Spain7 is able to evade early innate immune responses, which could explain its high virulence *in vivo* (García-Sánchez et al., under review).

Little is known about the parasite effectors implicated in modulation of the host immune response against *N. caninum*. ROP5, ROP16, GRA6, and GRA7 have been postulated as *N. caninum* virulence factors based on a mouse bioassay (Ma et al., 2017a,b; Nishikawa et al., 2018; Fereig et al., 2019). However, their potential in modulating host cell functions has not been demonstrated in the natural bovine host of *N. caninum*.

Although *N. caninum* seems to be highly conserved genetically, an important biological diversity has been demonstrated between isolates (Beck et al., 2009). Proteomic and transcriptomic analyses have emerged as very useful tools for study of the molecular mechanisms governing differences in virulence (Horcajo et al., 2018). However, to date, the only comparative study of *N. caninum* gene expression between isolates with different virulence in bovine target cells was carried out using trophoblast cells infected with Nc-Spain7 and Nc-Spain1H. The study reported different expression levels of genes involved in cell cycle, stress response and metabolic processes between the isolates, which could explain their biological differences (Horcajo et al., 2017). In the present work, the transcriptional profile of Nc-Spain7 and Nc-Spain1H isolates in boMØs was investigated to identify parasite effectors implicated in the differential ability of the isolates to modulate cellular processes related to the host cell immune response, which may suggest potential targets for control of this parasite.

## Materials and Methods

### Ethical Statements

Blood sampling and cow handling were conducted according to Spanish and EU legislation (Law 32/2007, concerning animals, their exploitation, transportation, experimentation and sacrifice; Royal Decree 53/2013 for the protection of animals employed in research and teaching; Directive 2010/63/UE, related to the protection of animals used for scientific goals). All procedures were approved by the Animal Welfare Committee of the Community of Madrid, Spain (permit number PROEX 236/17).

### Generation of Bovine Monocyte-Derived Macrophages

BoMØs were generated following the protocol described by García-Sánchez et al. (2019). Briefly, 900 ml of peripheral blood was collected from a healthy adult Holstein dairy cow that tested negative for infectious bovine rhinotracheitis virus (IBRV), bovine viral diarrhea virus (BVDV) and *N. caninum*. Histopaque 1077 (Sigma-Aldrich, USA) was used to separate peripheral blood mononuclear cells by density gradient, and isolation of monocytes was carried out by positive selection using anti-human CD14 antibody-conjugated microbeads (Miltenyi Biotec Ltd., USA). Monocytes were incubated at 37°C in a 5% CO_2_ atmosphere in medium containing 100 ng/ml recombinant bovine GM-CSF (Kingfisher Biotech Inc, USA). At day 5, boMØs were harvested, reseeded at 3 × 10^6^ cells/well in 6-well culture plates (P6) and incubated for 24 h prior to infection to minimize cellular stress due to the harvest procedure.

### Parasite Culture and Macrophage Infection

The *N. caninum* isolates Nc-Spain7 and Nc-Spain1H, which have shown important differences in virulence *in vitro* and *in vivo* models were used for MØ infection. Specifically, higher invasion and proliferation rates *in vitro* have been demonstrated for Nc-Spain7 than for Nc-Spain1H (Regidor-Cerrillo et al., 2011; Dellarupe et al., 2014; Jiménez-Pelayo et al., 2017). Higher parasite burden and more severe lesions, together with high rates of transplacental transmission and neonatal mortality were shown in pregnant mice infected with Nc-Spain7 (Regidor-Cerrillo et al., 2011; Dellarupe et al., 2014), whereas very low vertical transmission and neonatal mortality were observed in mice infected with Nc-Spain1H (Rojo-Montejo et al., 2009). In pregnant bovine models, 100% of transplacental transmission and fetal mortality was detected after Nc-Spain7 infection at early gestation (Caspe et al., 2012; Regidor-Cerrillo et al., 2014), and at least 50% of fetal mortality at mid-gestation (Almería et al., 2016; Jiménez-Pelayo et al., 2019a; Vázquez et al., 2019). However, Nc-Spain1H infection spared the fetus at early (Rojo-Montejo et al., 2009) and mid gestation (Jiménez-Pelayo et al., 2019a).

Tachyzoites of both isolates were routinely maintained in an MA-104 cell line culture as described previously (Regidor-Cerrillo et al., 2011), with the number of culture passages limited to fewer than 15 to reduce potential changes in virulence (Pérez-Zaballos et al., 2005). For boMØ infection, tachyzoites were harvested from 3 day-growth cultures, when at least 80% of the parasites were still in parasitophorous vacuoles, and purified with PD-10 Desalting Columns (G. E. Healthcare, UK) as described previously (Regidor-Cerrillo et al., 2011). To preserve the viability and invasion capacity of the tachyzoites, boMØs were inoculated with each isolate at a multiplicity of infection (MOI) of 3 within 1 h of parasite collection. At 8 h post infection (hpi), samples were recovered by scraping of cells in three P6 wells/condition (Nc-Spain7- and Nc-Spain1H-infected boMØs) followed by centrifugation for 10 min at 1,350 g. The obtained pellet containing 9 × 10^5^ cells was resuspended in 300 μl of RNA later (Thermo Fisher Scientific, Spain) and stored at −80°C. Three biological replicates were assessed, obtained in three independent experiments.

### RNA Extraction, RNA-Seq, and Data Computational Analysis

A Maxwell® 16 LEV simply RNA Purification Kit (Promega, USA) was used for total RNA extraction. RNA purity and concentration were determined at 260/280 nm using a NanoPhotometer Classic spectrophotometer (Implen, Germany). RNA integrity was assessed via electrophoresis on a 1% agarose gel stained with GelRed (Biotium, USA).

RNA-seq and computational analysis of the obtained data were performed as previously described (García-Sánchez et al., under review) with minimal modification. The quality and quantity of total RNA were assessed with a Bioanalyzer 2100 (Agilent, USA) and Qubit 2.0 (Thermo Fisher Scientific, Spain). The poly (A) + mRNA fraction was isolated from total RNA, and cDNA libraries were obtained according to Illumina's recommendations. The quantity of the libraries was determined via real-time PCR with a LightCycler 480 system (Roche, Germany) and the Bioanalyzer 2100. A High Sensitivity assay was used to assess their quality. Equimolar pooling of the libraries was performed prior to cluster generation in cBot (Illumina, USA). The pool of libraries was sequenced in an Illumina HiSeq 2000 sequencer (Illumina).

Data quality was assessed with the FastQC tool (http://www.bioinformatics.babraham.ac.uk/projects/fastqc). The raw paired-end reads were mapped against the *N. caninum* Liverpool strain genome provided by ToxoDB database version 28 (http://www.toxodb.org) using the TopHat2 v2.1.0 algorithm (Kim et al., 2013). Picard Tools (http://picard.sourceforge.net) was used to eliminate low quality reads. High quality reads were selected, assembled and identified via Bayesian interference through the algorithm proposed by Cufflinks v2.2.1 (Trapnell et al., 2010). Gene quantification was carried out using htseq_count 0.6.1p1 (Anders et al., 2015). For isoform quantification and differential expression, the Cufflinks method (Trapnell et al., 2010) was used.

### Differential Expression Determination and Functional Analyses

The statistical software R (R Studio Team, 2015) was used to determine the correlation between samples of the same condition prior to their acceptance as biological replicates by considering the whole transcriptome normalized by the size of the library.

Differential expression between sample groups was studied using the algorithm proposed by DESeq2 (Anders and Huber, 2010), with a binomial negative distribution for determination of the statistical significance (Love et al., 2014). Genes and isoforms were considered differentially expressed (DE) when they presented a Fold Change (FC) ≥2 and a false discovery rate (FDR)–adjusted (Benjamini and Hochberg, 1995) *p*-value (p adj) ≤ 0.05.

For functional analyses of *N. caninum* genes, an orthology analysis was carried out using the *T. gondii* database (http://toxodb.org/toxo/, ToxoDB release 42) due to the more complete annotation of the *T. gondii* genome.

### Transcriptome Validation via RT-qPCR

Transcriptome validation analysis was carried out as previously described with minimal modification (García-Sánchez et al., under review). Briefly, three additional biological replicates were collected and prepared as described for RNA-seq analysis. cDNA was obtained from extracted RNA using a master mix SuperScript VILO cDNA Synthesis Kit (Invitrogen, UK). The expression levels of selected genes of interest were measured via quantitative real-time PCR (qPCR) using four serial dilutions (1:20, 1:80, 1:320 and 1:1280) of each sample and normalized to those of the housekeeping genes NcTUBα (NCLIV_058890) and NcSAG1 (NCLIV_033230). Primers used to amplify the target genes are listed in Supplementary Table S1. Reactions were performed in a 7500 Fast Real-Time PCR System (Applied Biosystems, USA) using Power SYBR PCR Master Mix (Applied Biosystems). Relative gene expression was calculated using the 2^−ΔΔ*Ct*^ method (Livak and Schmittgen, 2001) and by comparing the expression of Nc-Spain1H vs. Nc-Spain7.

## Results and Discussion

### Sequencing and Mapping Data Obtained From RNA-Seq Analysis

In total, 3 biological replicates from boMØs inoculated with Nc-Spain1H and 3 from boMØs inoculated with Nc-Spain7 were sequenced individually via RNA-seq. Over 50 million reads were obtained for each sample. Between 6 and 16% of reads were mapped against the *N. caninum* genome (ToxoDB release 28). The lack of degradation of the starting biological material and the absence of significant deviations in the sequencing processes was verified by data quality controls based on duplication studies and G+C content. Distribution analysis of normalized data showed a correct distribution of biological replicates. No outliers were identified. Table 1 shows the results obtained by sample in the sequencing process: number of reads generated, mapped reads against the *N. caninum* genome and splice reads (related to the capability of the system to detect isoforms and splicing events). A low percentage of mapped reads against the parasite observed was expected because the majority of RNA from the samples would belong to the bovine host cell. Nevertheless, 6,672 parasite genes were detected (RPKM >1 in at least 1 sample), indicating representation of the parasite transcriptome. Of these, 6,484 genes were identified using the *N. caninum* Liverpool strain genome from ToxoDB database, and 188 predicted genes were not identified. These were named with an identification number preceded by “XLOC” (Supplementary Table S2). These results suggest that the Nc-Liverpool genome, the only *N. caninum* genome currently publicly available (Reid et al., 2012) should be study in depth for better annotation. Thus, this study, together with other *N. caninum* high-throughput experimental analyses, could be used to reveal previously undescribed genes (Saha et al., 2002) and to improve annotation of the reference genome.

**Table 1:** Mapped and paired reads by sample against *N. caninum* genome.

**Sample^a^**	**Total reads**	**Mapped reads**	**Mapped reads (%)**	**High quality reads**	**High quality reads (%)**	**Splice reads**	**Splice reads (%)**
1	45,726,510	5,750,052	12.57	4,067,318	8.89	465,001	1.02
2	49,810,014	3,120,428	6.26	2,160,028	4.34	245,772	0.49
3	46,709,092	3,987,977	8.54	2,949,996	6.32	340,289	0.73
4	50,818,888	6,302,701	12.4	4,260,546	8.38	525,535	1.03
5	59,129,224	4,957,694	8.38	3,321,220	5.62	404,784	0.68
6	48,795,612	7,718,581	15.82	5,774,492	11.83	724,179	1.48

a *Samples 1-3 correspond to Nc-Spain1H- infected boMØs; samples 4-6 correspond to Nc-Spain7-infected boMØs*.

**Figure 1:**
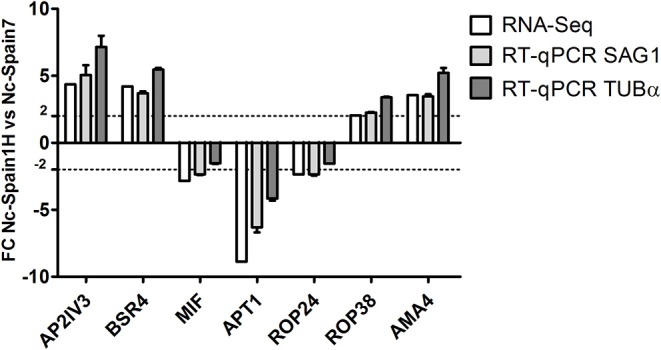
Validation of RNA-seq analyses via RT-qPCR. Bar graphs show Fold Change (FC) in *N. caninum* gene expression in Nc-Spain1H vs. Nc-Spain7; SAG1 and TUBα served as housekeeping genes.

Among the total *N. caninum* genes identified, 474 genes (7.31%) were DE between Nc-Spain1H and Nc-Spain7 isolates: 265 with higher expression in Nc-Spain7 and 209 with higher expression in Nc-Spain1H (Supplementary Table S2). Additionally, of the total genes identified and categorized as DE between isolates, 16 were exclusively expressed in Nc-Spain7, and 8 were exclusively expressed in Nc-Spain1H. Among the XLOC genes, 31 were DE, 16 were overexpressed in Nc-Spain1H, and 19 were overexpressed in Nc-Spain7. Three of these were exclusively expressed in the low virulence isolate and 1 in the high virulence isolate. Genes exclusively expressed by certain isolates should be taken into account because they may be related to the observed biological variability between isolates. Thus, further study of these genes as putative virulence factors should be considered in future research. RNA-seq results were validated by RT-qPCR for 7 genes DE between Nc-Spain7 and Nc-Spain1H (Figure 1).

It is noteworthy that the number of DEGs detected in our study is markedly higher than that reported in the transcriptomic analysis of bovine trophoblast cells infected with Nc-Spain7 and Nc-Spain1H isolates at 12 hpi (Horcajo et al., 2017). In that study, the first and until now the only comparative study of the transcriptional profile of two *N. caninum* isolates of different virulence in target cells of their natural host, 176 genes were found to be DE with FC ≥ 2 and p adj ≤ 0.05; 28 were highly expressed in Nc-Spain7, and 148 were highly expressed in Nc-Spain1H. We found that 25 genes were DE with a similar fold change between isolates in both boMØs and trophoblast cells (Table 2). However, comparison of both studies also revealed that the gene expression profile of the isolates strongly differed depending on the infected host cell. This behavior has also been reported in *T. gondii* and has been associated with the high ability of the parasite to adapt to different niches (Swierzy et al., 2017). To date, the impact of the host cell type on *N. caninum* phenotype has not yet been elucidated. As described for *T. gondii, N. caninum* may sense diverse cellular microenvironments and respond showing an heterogeneous transcriptional response between the different cell types. Because trophoblasts and macrophages are important targets for the parasite in the bovine host, the genes that may be inherent to the isolate phenotype independent of the host cell might be related to the differences in pathogenesis reported for both isolates in cattle (Rojo-Montejo et al., 2009; Regidor-Cerrillo et al., 2014), specifically with differences in proliferation or immune response modulation between the isolates described in both cell lines (Jiménez-Pelayo et al., 2017, 2019b; García-Sánchez et al., 2019). Thus, they should be considered for functional validation in future studies.

**Table 2:** DEG in the comparison Nc-Spain1H vs. Nc-Spain7 in infected bovine macrophages and trophoblast cells.

***Nc* gene id**	***Nc* description**	***Tg* gene id**	***Tg* description**	**FC^a^**	**padj^a^**	**FC^b^**	**padj^b^**
NCLIV_046430	Putative protein kinase	TGME49_226540	Protein kinase	2.48	9.2E-27	7.66	7.34E-03
NCLIV_019730	Hypothetical protein	TGME49_280420	HEAT repeat-containing protein	8.54	4.57E-133	16.16	1.45E-02
NCLIV_044060	Hypothetical protein	TGME49_305590	ABC transporter transmembrane region domain-containing protein	2.56	3.77E-35	3.61	4.50E-04
NCLIV_052500	Hypothetical protein	TGME49_215590	Flavoprotein subunit of succinate dehydrogenase	2.42	2.54E-06	3.56	3.17E-02
NCLIV_005970	Putative oocyst wall protein	TGME49_222940	Hypothetical protein	2.31	6.51E-23	5.29	4.23E-03
NCLIV_056890	Hypothetical protein	–		2.36	8.25E-10	3.34	4.50E-04
NCLIV_038440	Hypothetical protein	TGVEG_279350	Putative transmembrane protein	8.48	7.49E-101	9.63	4.50E-04
NCLIV_036640	Hypothetical protein	TGME49_269950	Hypothetical protein	5.39	1.12E-122	10.55	4.50E-04
NCLIV_038280	Hypothetical protein	TGME49_200450	Hypothetical protein	4.75	8.43E-97	8.56	4.50E-04
NCLIV_037980	Hypothetical protein	TGME49_268220	Hypothetical protein	3.63	7.48E-44	2.79	4.50E-04
NCLIV_069090	Hypothetical protein	–		3.02	6.81E-03	4.07	5.56E-03
NCLIV_049520	Hypothetical protein	TGME49_234380	Hypothetical protein	2.78	7.30E-04	5.64	4.50E-04
NCLIV_033310	Hypothetical protein	–		2.50	2.40E-34	6.36	4.50E-04
NCLIV_013400	Hypothetical protein	TGRUB_213445A	Hypothetical protein	2.00	1.69E-21	2.19	4.50E-04
NCLIV_065390	Bifunctional dihydrofolate reductase-thymidylate synthase, related	TGME49_249180	Bifunctional dihydrofolate reductase-thymidylate synthase	−2.72	4.67E-17	−2.04	2.51E-02
NCLIV_065280	Proliferating cell nuclear antigen, related	TGME49_247460	Proliferating cell nuclear antigen PCNA1	−3.14	1.04E-23	−2.81	4.22E-02
NCLIV_006510	Putative TCP-1/cpn60 family chaperonin	TGME49_297500	T-complex protein 1 eta subunit	−2.31	1.50E-12	−2.30	2,27E-02
NCLIV_025530	Putative TPR domain-containing protein	TGME49_262100	Tetratricopeptide repeat-containing protein	−2.70	3.42E-20	−2.28	4.57E-02
NCLIV_062520	3-ketoacyl-(Acyl-carrier-protein) reductase, related	TGME49_217740	3-ketoacyl-(acyl-carrier-protein) reductase	−9.03	9.13E-27	−5.02	3.27E-02
NCLIV_063860	Putative thioredoxin	TGME49_247350	Thioredoxin domain-containing protein	−2.52	1.78E-25	−2.11	4.50E-04
NCLIV_014020	Peroxiredoxin-2E-1, related	TGME49_286630	Redoxin domain-containing protein	−6.06	2.83E-33	−3.18	3.56E-02
NCLIV_007770	Putative Rhoptry kinase family protein, truncated (incomplete catalytic triad)	TGME49_253330	Rhoptry kinase family protein, truncated (incomplete catalytic triad)	−4.75	4.63E-104	−2.40	4.50E-04
NCLIV_068850	Unspecified product	TGME49_252360^c^	Rhoptry kinase family protein ROP24 (incomplete catalytic triad)	−2.47	1.63E-35	−2.79	4.50E-04
NCLIV_052780	Putative penicillin amidase domain-containing protein	TGME49_275320	Penicillin amidase	−2.87	1.07E-31	−2.53	4.50E-04
NCLIV_047150	Hypothetical protein	TGME49_225560	Hypothetical protein	−4.16	2.96E-74	−2.35	4.50E-04

a *Corresponds to infected bovine macrophages samples*.

b *Corresponds to infected trophoblast cells (F3 cell line) samples*.

c *No synthenic*.

A further analysis of the 474 DEGs between the isolates was undertaken. Of these, 290 (61.18%) were annotated as hypothetical proteins in the *N. caninum* database. Orthological analysis with the *T. gondii* database revealed that 22 of these 290 genes do not have orthologs in *T. gondii*. A total of 105 of the remaining genes were also annotated as hypothetical proteins for their orthologous in *T. gondii* database. Our study provides evidence that these hypothetical genes are actually expressed during the intracellular development of the parasite and thus may be important for the biology of the parasite and be related with differences in virulence between Nc-Spain7 and Nc-Spain1H.

**Figure 2:**
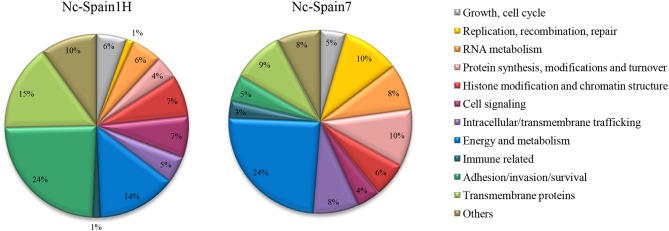
Functional classification of *N. caninum* genes differentially expressed between isolates. Pie charts showing the percentage of genes for each functional category relative to the total overexpressed genes for each isolate.

**Figure 3:**
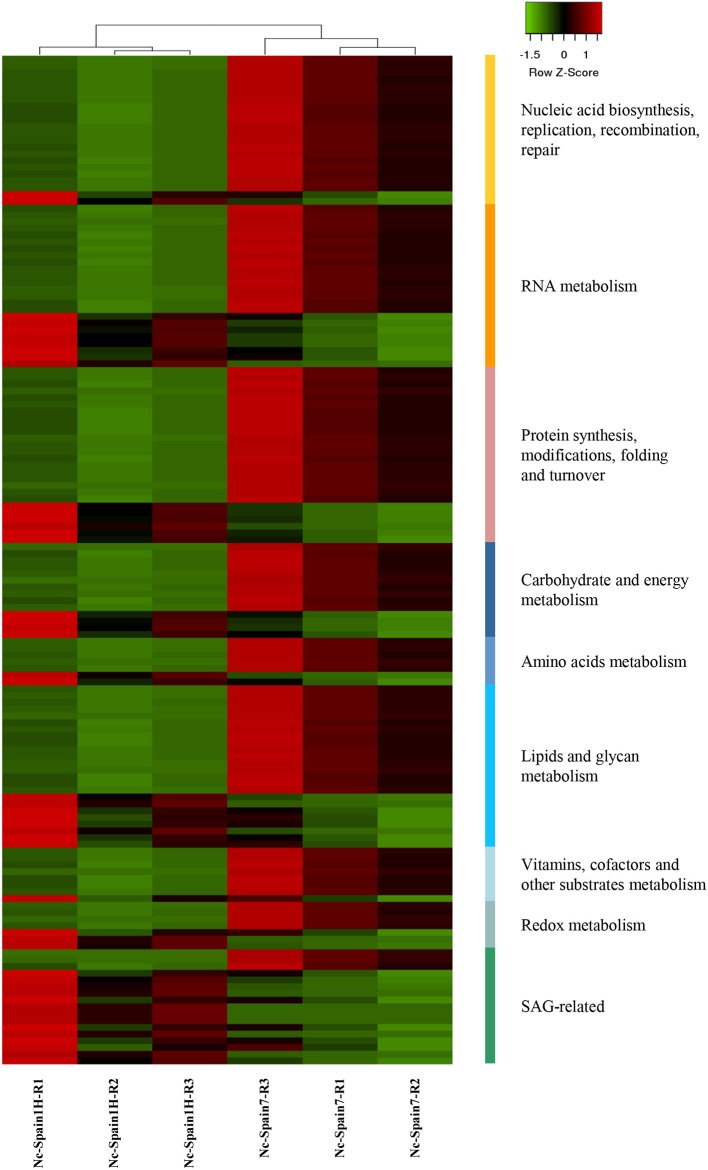
Clustering of *N. caninum* genes differentially expressed between isolates. Heatmap showing a selection of *N. caninum* DEGs with Z-scores (row) based on expression data of three replicates (R1-R3) of boMØs challenged for 8 h with Nc-Spain1H and Nc-Spain7 tachyzoites. The heatmap was generated using Heatmapper (http://www2.heatmapper.ca). The genes are grouped according to functional categories and were clustered using the Pearson computing distance method.

The 474 DEGs between both isolates in bovine macrophages were functionally classified and are listed in Supplementary Table S3. The most representative subsets are presented in Figures 2, 3, where a very different expression pattern is shown for the two isolates. Because classification of genes annotated as hypothetical proteins for *N. caninum* has been carried out by homology with *T. gondii* and important differences in the biology of both sp4ecies do exist, future validation is necessary to experimentally prove their functions.

### Host Cell Adhesion-Related SRS and MIC Genes Were Highly Expressed in Nc-Spain1H and Genes Related to Gliding Motility Were Highly Expressed in Nc-Spain7

Adhesion of *N. caninum* to the host cell prior to invasion initially occurs through a low-affinity and reversible interaction mediated by the tachyzoite surface antigen molecules. This is followed by establishment of a more stable adhesion that requires secretion of proteins from micronemes (Hemphill et al., 2006). A total of 14 surface antigen (SAG)-related sequences were overexpressed in Nc-Spain1H, and only 3 were overexpressed in Nc-Spain7. In addition, 2 microneme proteins, MIC12 (NCLIV_069310) and MIC14 (NCLIV_061910), showed higher expression in Nc-Spain1H but none showed higher expression in Nc-Spain7. Other proteins that could also be related to parasite-host cell adhesion are those containing epidermal grow factor (EGF) and thrombospondin (TSP) domains (Hemphill et al., 2006, 2013), among which 3 genes (NCLIV_036660, NCLIV_011000, and NCLIV_001350) were overexpressed in Nc-Spain1H, and only 1 gene (NCLIV_003470) was overexpressed in Nc-Spain7. NCLIV_001350 corresponds to the orthologous apical membrane antigen 4 (AMA4) of *T. gondii*. TgAMA4 has been described as not only being involved in attachment but also in internalization of the parasite through interaction with the rhoptry neck protein RON2L1, which maintains the moving junction structural integrity required for tachyzoite entry (Lamarque et al., 2014). In fact, RON2L1 (NCLIV_001400) is also overexpressed in Nc-Spain1H.

The higher expression of genes associated with adhesion in Nc-Spain1H is striking, because *in vitro* studies with different cell lines have shown that Nc-Spain1H has a lower invasion rate than Nc-Spain7 (Regidor-Cerrillo et al., 2011; Jiménez-Pelayo et al., 2017), including boMØs (García-Sánchez et al., 2019). However, a similar result was obtained in a transcriptomic analysis of these isolates in bovine trophoblast cells (Horcajo et al., 2017), in which 9 SAG-related sequences and 22 proteins from micronemes were found to be overexpressed in Nc-Spain1H, and none were overexpressed in Nc-Spain7. In further support of our results, a proteome analysis of Nc-Spain7 and Nc-Spain1H cultured in MARC-145 cells revealed that surface antigen and microneme protein abundance differed between isolates throughout the tachyzoite lytic cycle (Horcajo et al., 2018). It is worth noting that the adhesion function of SRS and MIC proteins has been determined by homology with *T. gondii*, but to the best of our knowledge, this function has only been demonstrated in *N. caninum* for SAG1, SRS2, MIC1-4 and 17 (Hemphill et al., 2006; Sohn et al., 2011), none of which were differentially expressed between our isolates. In addition, mechanisms other than adhesion are involved in invasion and may be enhanced in Nc-Spain7. With regard to this connection, previous studies in trophoblast cell lines (Horcajo et al., 2017; Jiménez-Pelayo et al., 2017), have associated the higher invasion capacity of Nc-Spain7 with enhanced expression of gliding-associated proteins by the isolate in the trophoblast cell line. For Nc-Spain7, enhanced expression of two glideosome genes has been reported: the microtubule-associated protein SPM1 (NCLIV_024420), which colocalizes with the subpellicular microtubules and whose deletion decreases tachyzoite fitness in *T. gondii* (Tran et al., 2012); and a putative actin-like family protein (ALP, NCLIV_037160). Although ALPs may be implicated in actin-based gliding motility, vesicle transport and transcriptional regulation through chromatin remodeling are other functions attributable to these proteins (Gordon and Sibley, 2005).

Apart from their putative relationship with adhesion to the host cell, SRS proteins have been recognized as the primary surface antigens and may determine the immunogenic potential of the isolates (Lekutis et al., 2001). In this connection, SRS genes are overexpressed in Nc-Spain1H and may be related to its capacity to induce a higher proinflammatory response by boMØs infected *in vitro* than Nc-Spain7 (García-Sánchez et al., 2019). A study of these antigen determinants differentially expressed between Nc-Spain7 and Nc-Spain1H (Supplementary Table S3) may help to determine why Nc-Spain1H seems to be more efficiently detected by the host immune system (Jiménez-Pelayo et al., 2017; García-Sánchez et al., 2019).

### Rhoptry Proteins Were Differentially Expressed Between the Isolates

Once *N. caninum* attachment to the host cell is achieved, rhoptries are released to form the moving junction, which is a structure that facilitates parasite entry and formation of the parasitophorous vacuole. Then, dense granules secrete proteins to modify the parasitophorous vacuole, enabling intracellular survival of the parasite. These organelles also inject proteins into the cytosol of the host cell to modulate their functions, ensuring success of the infection (Sohn et al., 2011).

For Nc-Spain7, genes encoding for two predicted rhoptry proteins were highly expressed: a predicted member of the rhoptry-kinase-like family (ROPKL) subfamily BPK1 (NCLIV_007770), and a member of the rhoptry kinase family ROP20 (NCLIV_068850), specific for *N. caninum* and orthologous but not syntenic to *T. gondii* ROP24. Higher NCLIV_007770 expression was also observed for Nc-Spain7 in the comparative transcriptomic analysis of infected bovine trophoblast cells (Horcajo et al., 2017). However, of special interest is NCLIV_068850, whose enhanced expression in Nc-Spain7 has been demonstrated to be maintained for tachyzoite cell cycle progression regardless of the host cell (Horcajo et al., 2017, 2018).

Regarding Nc-Spain1H, in addition to RON2L1, mentioned above, higher expression was observed for the predicted rhoptry kinase subfamily gene ROP30 (NCLIV_001950), an ortholog to *T. gondii* ROP4/ROP7, and two predicted lineage-specific rhoptry kinases of the subfamily ROPK-Eten1 (NCLIV_017420 and NCLIV_068890), which are orthologs to the *T. gondii* gene locus ROP19/ROP28/ROP38 (Kemp et al., 2013). Peixoto et al. (2010) pointed to ROP38 and ROP4/ROP7 as likely to be particularly important in the biology of *T. gondii*. ROP38, highly expressed by low virulence *T. gondii* strains and during tachyzoite to bradyzoite conversion, down-modulates genes associated with MAPK signaling in the host cell and with regulation of apoptosis and proliferation (Peixoto et al., 2010). It has also been shown that knockout of ROP38 leads to increased invasion and proliferation of *T. gondii*, and infection of mice with ROP38-deficient parasites resulted in lower levels of IL-18 and IL-1β production, which was related to a decrease in *T. gondii* profilin expression relative to wild-type parasites (Xu et al., 2018). In addition, ROP38 is among the most deeply regulated genes in the genome of the parasite, as the expression level of ROP38 is highly variable among *Toxoplasma* strains (Melo et al., 2013), and evidence of evolutionary selection at the species and population level has been reported (Khan et al., 2009; Peixoto et al., 2010). Its ancestor gene was independently triplicated in *N. caninum* and *T. gondii*. Notably, expanded genes have been linked to virulence, immune evasion or host range in *T. gondii* and other pathogens, such as *Plasmodium* spp. (Adomako-Ankomah et al., 2014). ROP4/ROP7, which are among the most highly expressed genes in the genome of *T. gondii*, may be functionally relevant based on studies showing that expression levels are important for virulence (Saeij et al., 2007; Peixoto et al., 2010).

Interestingly, no dense granule or other rhoptry-encoding genes recognized as virulence factors in *T. gondii* or *N. caninum* were found to be DE between Nc-Spain7 and Nc-Spain1H isolates. It is important to emphasize that characterization of *N. caninum* ROP and GRA proteins is limited, and host regulation in many cases does not correlate with the respective *T. gondii* orthologs. Such is the case with NcROP16, which in *N. caninum* activates STAT3, thereby promoting host cell apoptosis and enhancing the pathogenicity of the parasites (Jensen et al., 2011; Ma et al., 2017a), whereas TgROP16 secreted by type I and III strains activates STAT6 in MØs, leading to alternative activation of these cells (Jensen et al., 2011). More importantly, *T. gondii* and *N. caninum* virulence factors have been identified in mice, and it has been demonstrated that virulence can be host-dependent (Sánchez-Sánchez et al., 2018), likely due to the marked differences in the immune system observed between species that may determine the host-pathogen interplay.

### Bradyzoite-Related Genes Showed Higher Expression in Nc-Spain1H

Bradyzoites represent the quiescent stage of the parasite, triggered by stress caused by the host immune response (Hemphill et al., 2006). The transcriptomic analysis of *N. caninum* isolates in boMØs revealed a higher expression of 2 *N. caninum* bradyzoite-specific genes in the Nc-Spain1H isolate: the surface proteins SAG4 (NCLIV_019580) and BSR4 (NCLIV_010030) (Fernández-García et al., 2006; Risco-Castillo et al., 2007; Horcajo et al., 2017, 2018). In addition, our results showed differential expression of other genes that have been shown to be related to the tachyzoite-to-bradyzoite conversion in *T. gondii*, such as AP2 transcription factors (Huang et al., 2017). Nc-Spain1H showed overexpression of two AP2 factors known to be up-regulated during bradyzoite development in *T. gondii:* AP2IV-3 (NCLIV_010930), whose deletion in *T. gondii* results in a lower capacity to form tissue cysts (Hong et al., 2017); and AP2X-10 (NCLIV_052260), which is highly expressed by low-virulence *T. gondii* isolates in murine MØs (Melo et al., 2013). In contrast, Nc-Spain7 showed enhanced expression of two transcription factors related to the tachyzoite stage: AP2XII-2 (NCLIV_062490) and AP2IV-4 (NCLIV_011080) (Huang et al., 2017; Radke et al., 2018). AP2IV-4 is necessary for suppression of bradyzoite surface antigens and cyst wall proteins in the tachyzoite stage and is exclusively expressed in the tachyzoite division cycle (Radke et al., 2018). Previous results from a murine *T. gondii* infection model determined that tachyzoites lacking this gene expressed bradyzoite antigens at the wrong time, stimulating a strong immune response by monocytes. This response successfully eliminated the parasite, preventing tissue cyst formation in mouse brain (Radke et al., 2018). Thus, it would be interesting to assess the implication of AP2IV-4 in the observed differential response induced by Nc-Spain1H and Nc-Spain7 in boMØs and in their improved capacity to evade the innate immune response (García-Sánchez et al., under review).

Other genes overexpressed in the Nc-Spain1H isolate and with higher expression during bradyzoite development reported in *T. gondii* encode for H2A histone proteins (NCLIV_025910), which are implicated in transcription regulation and DNA repair and whose enhanced expression is likely associated with DNA damage resulting from oxidative stress (Dalmasso et al., 2009), and a cAMP-dependent protein kinase (PKA, NCLIV_004220), which is involved in cell cycle regulation and stress response (Wei et al., 2013; Pittman et al., 2014). Overexpression of bradyzoite-specific genes by Nc-Spain1H has been previously described and related to a prebradyzoite stage in which the parasite cell cycle shifts toward slower growth (Horcajo et al., 2018). Finally, two putative oocyst wall proteins (NCLIV_003900 and NCLIV_005970) exhibited higher expression in Nc-Spain1H.

### Higher Expressions of Genes Involved in Parasite Growth Were Found for the Virulent Isolate Nc-Spain7

Increased expression of genes involved in DNA replication, RNA metabolism, protein synthesis, cell division and energy production was observed in Nc-Spain7, which is consistent with the active parasite replication and higher growth rate of Nc-Spain7 shown *in vitro* in different studies (Regidor-Cerrillo et al., 2011; Jiménez-Pelayo et al., 2017; García-Sánchez et al., 2019).

### Nucleic Acid Biosynthesis, Replication, Recombination, and Repair

Higher expression of four genes related to purine and pyrimidine biosynthesis and salvage were observed in Nc-Spain7. We also found enhanced expression of DNA polymerases, origin recognition complexes, DNA replication licensing factors, a DNA topoisomerase, and several other genes encoding proteins associated with DNA replication (Supplementary Table S3), such as orthologs to the two putative proliferating cell nuclear antigens of *T. gondii*, PCNA1 (NCLIV_065280) and PCNA2 (NCLIV_010140). PCNA1, likely the major replisomal PCNA in *T. gondii* (Guerini et al., 2005), is one of the 11 genes up-regulated in Nc-Spain7 during infection of both boMØs and trophoblast cells (Horcajo et al., 2017).

### Histone Modification, Chromatin Structure, and Microtubule Dynamics

Enhanced expression of genes related to chromatin assembly and chromosome condensation was also shown for Nc-Spain7. This may reflect the need of proliferating tachyzoites to maintain active chromatin organization dynamics to accompany DNA replication (Parthun, 2007). The putative HU protein (NCLIV_045430) is a histone-like protein with fundamental roles in transcription, replication initiation, and DNA repair. In *T. gondii*, this protein localizes to the apicoplast and is required for genome maintenance and inheritance (Reiff et al., 2012). Because the apicoplast is unique to apicomplexan parasites and is not found in the host, proteins associated with this organelle are considered good target candidates for pathogen-specific drugs (Reiff et al., 2012).

Interestingly, six genes encoding dynein heavy chains and one encoding a dynein intermediate chain were found to be overexpressed in Nc-Spain1H. Dyneins are microtubule-associated proteins that function as motors for several processes, such as axoneme beating, organelle transport, spindle function, and centrosome assembly (Morrissette, 2015). *Neospora caninum* dyneins have been poorly studied. To the best of our knowledge, only the cytoplasmic dynein LC8 light chain 2 has been characterized and has been found to be related to virulence through regulation of host immunity (Cao et al., 2019). Future research is necessary to unravel the specific functions of the dynein proteins up-regulated in the low-virulence isolate.

### RNA Metabolism, Protein Synthesis, and Turnover

Thirty-six genes related to RNA metabolism and protein synthesis, modification, folding and turnover were also highly expressed in the high-virulence isolate (Supplementary Table S3) vs. 14 in the low-virulence isolate. Among these genes, we identified two RNA pseudouridine synthases (NCLIV_020280 and NCLIV_048340). Pseudouridine synthases catalyze the conversion of the RNA base uridine to pseudouridine. Although the role of pseudouridylation of RNA has been poorly studied, pseudouridylation seems to confer an important selective advantage in a natural biological context. It is known that mutation of pseudouridine synthases in *Escherichia coli* and *Saccharomyces cerevisiae* results in a slow-growth phenotype, and a *T. gondii* pseudouridine synthase has been discovered to be important in tachyzoite-to-bradyzoite differentiation (Charette and Gray, 2000; Anderson et al., 2009).

Nc-Spain7 showed enhanced expression of six genes related to the ubiquitin-proteasome system, three of them encoding proteasome beta subunits (NCLIV_048880, NCLIV_057270, and NCLIV_061460), one ortholog to the *T. gondii* NEDD8-activating enzyme E1 catalytic subunit (NCLIV_040320), one putative SUMO activating enzyme (NCLIV_011590), and cullin-associated NEDD8-dissociated protein 1 (NCLIV_052060). Proteasomes play essential roles in parasite biological processes, such as cell differentiation, cell cycle progression, proliferation, and encystation and have been suggested as virulence factors (Munoz et al., 2015). The proteasome system also seems to be an attractive drug target because inhibitors of this protein complex have been shown to diminish infectivity and block intracellular growth and replication of *T. gondii in vitro* (Shaw et al., 2000; Paugam et al., 2002), and differences between the proteasomes of mammals and parasites have been observed (Munoz et al., 2015).

### Carbohydrate, Amino Acid, and Fatty Acid Metabolism

A great number of genes related to *N. caninum* metabolism, primarily involved in carbohydrate, amino acid and fatty acid metabolism, exhibited higher expression in the Nc-Spain7 isolate. Similar results were obtained by Horcajo et al. (2017), through transcriptomic analysis of both isolates in trophoblast cells, where it was hypothesized that more energy is consumed by an isolate with a higher growth rate, and thus, a more activate metabolism may be present in Nc-Spain7 than in Nc-Spain1H.

With respect to carbohydrate metabolism, we found higher expression of ten genes involved in obtaining energy via glycolysis, pyruvate metabolism and starch and galactose metabolism pathways in Nc-Spain7. Regarding lipid metabolism, eight genes of the apicoplast-localized FAS II system were highly expressed in this isolate vs. only one in the Nc-Spain1H isolate. In *T. gondii, de novo* fatty acid synthesis via FAS II is essential for parasite growth and virulence (Mazumdar et al., 2006), and because this pathway is parasite-specific, its components represent key targets for the development of selective drugs (Coppens, 2013; Wu et al., 2018). One of these targets may be the apicoplast triosephosphate translocator APT1, whose ortholog in *N. caninum* was highly expressed in the high-virulence isolate in this and previous studies (Horcajo et al., 2018). TgAPT1 is not only required for the FASII pathway, but it also delivers carbon for another anabolic process in the apicoplast, the DOXP pathway. In addition, it has a role in indirectly supplying the apicoplast with ATP and redox equivalents (Brooks et al., 2010).

Another interesting gene related to fatty acid metabolism that was highly expressed by Nc-Spain7 is a putative patatin-like phospholipase (PLP, NCLIV_033980). PLPs are found in many pathogens and have been associated with host-cell interactions and immune evasion. Multiple PLPs are conserved across the subphylum Apicomplexa, suggesting a critical role for these enzymes in parasites, and several *T. gondii* PLPs are currently under investigation for their potential importance in parasite virulence (Wilson and Knoll, 2018). Prominent among them is TgPL1, which protects tachyzoites from nitric oxide-related degradation in activated MØs (Tobin Magle et al., 2014).

### Genes Involved in Redox Homeostasis Were Differentially Expressed Between Isolates

Four genes highly expressed by Nc-Spain7 were identified as belonging to the redox metabolism pathway: an *N. caninum* ortholog (NCLIV_055730) to *T. gondii* apicoplast-associated thioredoxin Atx1 (NCLIV_055730), glutaredoxin (NCLIV_045930), thioredoxin (NCLIV_063860) and peroxiredoxin (NCLIV_014020). In addition, these two last enzymes are also highly expressed in the high-virulence isolate Nc-Spain7 according to a previous transcriptomic analysis of bovine trophoblast cells (Horcajo et al., 2017).

TgATrx1 is a redox-regulated enzyme essential for the parasite. It is involved in endoplasmic reticulum-to-apicoplast trafficking and contributes to apicoplast biogenesis. Due to its importance in parasite biology, the potential of apicoplast thioredoxins as drug targets has been suggested (Biddau et al., 2018). Antioxidant enzymes permit apicomplexan parasites to address oxidative levels inside the host cells and seem especially important in regard to intracellular survival in MØs, which use reactive oxygen species to kill pathogens (Bosch et al., 2015). Thus, their enhanced expression may confer an advantage to the high-virulence isolate. In fact, higher parasite survival rates accompanied by lower intracellular ROS levels in boMØs infected by this isolate have been described previously (García-Sánchez et al., 2019).

### Immune Response Modulation-Related Genes Were Found to Be Differentially Expressed in Nc-Spain1H and Nc-Spain7

Interestingly, a dichotomy was found in the gene expression profiles of Nc-Spain 7 and Nc-Spain1H regarding potential immunomodulation functions. Genes potentially implicated in induction were found to be up-regulated in Nc-Spain1H, whereas genes involved in evasion of immune responses were found to be up-regulated in Nc-Spain7.

In addition to SRS genes, overexpressed in Nc-Spain1H as mentioned above, higher expression of a gene encoding a putative tryptophan-rich protein belonging to the Pv-fam-a family (NCLIV_004020) was shown for the low-virulence isolate. This family of immunogenic proteins has been characterized in malaria human and rodent parasites and been proposed as candidate antigens for potential vaccines (Wang et al., 2015).

Enhanced expression of the proteophosphoglycans (PPGs) PPG3 (NCLIV_005160), PPG4 (NCLIV_020320) and PPG5 (NCLIV_065610) was found in Nc-Spain7. These surface-coating molecules have been associated with immune evasion in *Leishmania* spp due to their ability to modulate MØ function during early infection by inhibiting TNF-α production. The ability of secreted PPGs to induce complement activation has been related to prevention of the opsonization of the parasite, and may contribute to the lesion development and pathology caused by *Leishmania mexicana*. In addition, the possibility of *Leishmania major* PPGs involvement in suppression of IL-12 production by dendritic cells has been also suggested (Peters et al., 1997; Depledge et al., 2009; Favila et al., 2015).

Glycosylphosphatidylinositols (GPIs) of *N. caninum* have recently been demonstrated to be secreted in supernatant and likely recognized by TLR2 and TLR4, are able to modulate APC immune responses. Interestingly, host-cell modulation seems to differ depending on the host origin of APCs. *Neospora caninum* GPIs have been shown to induce production of the proinflammatory cytokines TNF-α, IL-1β, and IL-12 in murine macrophages and dendritic cells. However, bovine PBMCs showed reduced levels of IL-12p40 and MHC II in response to GPIs (Débare et al., 2019). In the present study, three genes involved in the GPI biosynthetic pathway [orthologs to *T. gondii* mannosyltransferase (NCLIV_004260), N-acetylglucosaminyl phosphatidylinositol deacetylase (NCLIV_028260) and a PGAP1 family protein (NCLIV_054430)] were highly expressed by Nc-Spain7.

Nc-Spain7 also showed up-regulation of *N. caninum* macrophage migration inhibitory factor (MIF, NCLIV_042400). MIF homologs have been reported in several protozoan parasites, including *T. gondii, Plasmodium* spp, *Eimeria* spp and *Leishmania* spp and have been suggested to play a role in immune evasion (Qu et al., 2013; Sommerville et al., 2013).

In addition, the virulent isolate expressed higher levels of two genes that may be involved in dissemination and transmission: a putative cyclophilin (NCLIV_015405) and a calreticulin family member (NCLIV_054410). *Neospora caninum* molecules from the cyclophilin family have been shown to work as chemokine-like proteins by inducing chemoattraction of immune cells, which may consequently enhance their invasion by the parasites (Mineo et al., 2010). Because we have previously shown the ability of *N. caninum* to survive in boMØs and induce a hypermigratory phenotype in these cells upon infection (García-Sánchez et al., 2019), the higher expression of cyclophilin by Nc-Spain7 may potentially be related to the increased dissemination of this isolate found *in vivo* (Collantes-Fernández et al., 2012). Calreticulin is an endoplasmic reticule-resident chaperone involved in protein folding. In trypanosomatids, it seems to help in establishment of infection by modulating the host complement system (Ramakrishnan and Docampo, 2018). In addition, alteration of the function of this chaperone in *Leishmania donovani* results in a lower survival rate in MØs, and interestingly, *Trypanosoma cruzi* calreticulin likely facilitates placental infection by interacting with the maternal classical complement component C1, which would bridge it with the fetal calreticulin in placental tissues (Castillo et al., 2013).

The roles of these proteins in *N. caninum* pathogenesis, particularly with regard to differences in the proinflammatory response induced by the isolates during infection *in vitro* (García-Sánchez et al., 2019) and transmission found *in vivo* (Rojo-Montejo et al., 2009; Regidor-Cerrillo et al., 2014), require further investigation.

## Concluding Remarks

Intraspecific variations in the biological behavior of *N. caninum* isolates have been widely described and associated with differences in virulence. In previous studies, we demonstrated that Nc-Spain7 and Nc-Spain1H isolates, which exhibit marked differences in virulence, show different abilities to invade, survive and proliferate in boMØs and modulate the cell response in different manners. The Nc-Spain1H isolate, despite showing a lower proliferation and survival rate, induces higher expression of genes involved in pathogen recognition, chemotaxis and proinflammatory and regulatory cytokine release, which may result in key differences in the immune responses generated by the host against the isolates. Here, we describe that these differences are connected with specific gene expression profiles in the tachyzoite stage. Specifically, bradyzoite stage-related genes and genes encoding for surface antigens were among the primary up-regulated genes in the Nc-Spain1H isolate, whereas Nc-Spain7 showed enhanced expression of genes involved in parasite growth and survival in activated MØs.

Further studies are necessary to determine the virulence factor potential of the proteins identified to be differentially expressed between the isolates and determine whether these proteins may be implicated in parasite fitness and immune response modulation.

## Data Availability Statement

The datasets generated for this study can be found in the NCBI Sequence Read Archive under the identifier PRJNA552526.

## Ethics Statement

The animal study was reviewed and approved by Animal Welfare Committee of the Community of Madrid, Spain (permit number PROEX 236/17).

## Author Contributions

LO-M, JR-C, and EC-F conceived the study. PH participated in its design. MG-S wrote the manuscript and interpreted the results, with discussion input from LJ-P, PH, JR-C, EC-F, and LO-M. MG-S and LJ-P performed the experiments. All authors read and approved the final manuscript.

### Conflict of Interest

The authors declare that the research was conducted in the absence of any commercial or financial relationships that could be construed as a potential conflict of interest.
