# Supplementary material for: Gene Expression Profiling of Neospora caninum in Bovine Macrophages Reveals Differences Between Isolates Associated With Key Parasite Functions
Source: Front Cell Infect Microbiol. 2019 Oct 15;9:354. doi: 10.3389/fcimb.2019.00354 (PMC6803445; doi:10.3389/fcimb.2019.00354)
Supplement: Supplementary file 1 [file Table_1.DOCX]

**Supplementary Table S1**: Sequences of primers used for transcriptomic validation by RT-qPCR

| **Target** | **Ortholog TgME49** | **Sequences (5' to 3')** | **Product size (bp)** | **References** |
| --- | --- | --- | --- | --- |
| NCLIV_042400 | TGME49_290040 | CCGACCCGTGTGCGTACATT | 132 | This study |
| NcMIF | TgMIF | TGGTGTAGATGCGGTTTTTGGA |  |  |
| NCLIV_026210 | TGME49_261070 | GTCATCATGGCGTCAGTCAC | 154 | This study |
| Hypothetical protein | TgAPT1 | TGTTCGCAGACGACAGATTC |  |  |
| NCLIV_017420 | TGME49_242110 | AGTGAGTCTATTCGACGACGA | 169 | This study |
| Unspecified product | TgROP38 | AGCCACCTGCGTTCGCTGTCT |  |  |
| NCLIV_010930 | TGME49_318610 | AGAACAAGCGGACAACTTGGT | 202 | This study |
| Unspecified product | TgAP2IV-3 | GAAGTCGTCGAGATCCACTTC |  |  |
| NCLIV_001350 | TGME49_294330 | ACGGTAACCAGCATTTCCAG | 195 | This study |
| Putative EGF-like domain containing protein | TgAMA4 | TTCTTCTGCGTCTTCCTCGT |  |  |
| NCLIV_068850 | TGME49_252360 | GGCGAACCTTCATAAGCAAC | 139 | Horcajo et al., 2018 |
| Unspecified product | TgROP24^a^ | AAGTAAATCGGGGGAGCAAC |  |  |
| NCLIV_010030 | TGME49_320230 | CCATGAAGGACTCCAGAATCG | 101 | Risco-Castillo et al., 2007 |
| NcBSR4 | TgSRS15C | TCGCTGGAAACGCACATTTA |  |  |
| NCLIV_058890 | TGME49_316400 | GGTAACGCCTGCTGGGAG | 166 | Alaeddine et al., 2013 |
| NcTUBα | TgTUBA1 | GCTCCAAATCCAAGAAGACGCA |  |  |
| NCLIV_033230 | TGME49_233460 | CGGTGTCGCAATGTGCTCTT | 150 | Fernández-García et al., 2006 |
| SRS domain containing protein | TgSAG1 | ACGGTCGTCCCAGAACAAAC |  |  |

^a^ No syntenic

**References:**

Alaeddine, F., Hemphill, A., Debache, K. and Guionaud, C. (2013). Molecular cloning and characterization of NcROP2Fam-1, a member of the ROP2 family of rhoptry proteins in *Neospora caninum* that is targeted by antibodies neutralizing host cell invasion in vitro. *Parasitology* 140: 1033–1050. doi:10.1017/S0031182013000383

Fernández-García, A., Risco-Castillo, V., Zaballos, A., Álvarez-García, G., and Ortega-Mora, L. M. (2006). Identification and molecular cloning of the *Neospora caninum* SAG4 gene specifically expressed at bradyzoite stage*. Mol.Biochem.Parasitol.* 146: 89-97. doi:10.1016/j.molbiopara.2005.08.019

Horcajo, P., Xia, D., Randle, N., Collantes-Fernández, E., Wastling, J., Ortega-Mora, L., et al. (2018). Integrative transcriptome and proteome analyses define marked differences between *Neospora caninum* isolates throughout the tachyzoite lytic cycle*. J. Proteomics.* 180: 108-19. doi:10.1016/j.jprot.2017.11.007

Risco-Castillo, V., Fernández-García, A., Zaballos, A., Aguado-Martínez, A., Hemphill, A., Rodríguez-Bertos, A., et al. (2007). Molecular characterisation of BSR4, a novel bradyzoite-specific gene from *Neospora caninum. Int. J. Parasitol.* 37: 887-96. doi:10.1016/j.ijpara.2007.02.003
